# Association of *COMT* Val158Met polymorphism and breast cancer risk: an updated meta-analysis

**DOI:** 10.1186/1746-1596-7-136

**Published:** 2012-10-08

**Authors:** Xue Qin, Qiliu Peng, Aiping Qin, Zhiping Chen, Liwen Lin, Yan Deng, Li Xie, Juanjuan Xu, Haiwei Li, Taijie Li, Shan Li, Jinmin Zhao

**Affiliations:** 1 Department of Clinical Laboratory, First Affiliated Hospital of Guangxi Medical University, Nanning 530021, Guangxi, China; 2 Department of Obstetrics and Gynecology and Reproductive center, First Affiliated Hospital of Guangxi Medical University, Nanning, Guangxi, China; 3 Department of Occupational Health and Environmental Health, School of Public Health at Guangxi Medical University, Nanning, Guangxi, China; 4 Department of Orthopedic Trauma Surgery, First Affiliated Hospital of Guangxi Medical University, Nanning 530021, Guangxi, China; 5 Department of Clinical Laboratory, Baise City People's Hospital, Baise, Guangxi, China

**Keywords:** COMT, Polymorphism, Breast cancer, Meta-analysis

## Abstract

**Background:**

Catechol-O-methyltransferase (*COMT*) is one of the most important enzymes involved in estrogen metabolism and its functional genetic polymorphisms may be associated with breast cancer (*BC*) risk. Many epidemiological studies have been conducted to explore the association between the *COMT* Val158Met polymorphism and breast cancer risk. However, the results remain inconclusive. In order to derive a more precise estimation of this relationship, a large meta-analysis was performed in this study.

**Methods:**

Systematic searches of the PubMed, Embase and Cochrane Library were performed. Crude odds ratios (ORs) with 95% confidence intervals (CIs) were calculated to estimate the strength of the association.

**Results:**

A total of 56 studies including 34,358 breast cancer cases and 45,429 controls were included. Overall, no significant associations between the *COMT* Val158Met polymorphism and breast cancer risk were found for LL versus HH, HL versus HH, LL versus HL, recessive model LL versus HL+HH, and dominant model LL+HL versus HH. In subgroup analysis by ethnicity, source of controls, and menopausal status, there was still no significant association detected in any of the genetic models.

****Conclusion**:**

Our meta-analysis results suggest that the *COMT* Val158Met polymorphism may not contribute to breast cancer susceptibility.

**Virtual slides:**

The virtual slides(s) for this article can be found here: http://www.diagnosticpathology.diagnomx.eu/vs4806123577708417

## Introduction

Breast cancer is one of the most frequently occurring cancer and cancer-related deaths are highly prevalent worldwide, which has become a major public health challenge [1]. The mechanism of developing breast cancer is still unclear. It has been widely accepted that exposure to circulating estrogen may be important in the development of breast cancer. Since estrogen biosynthesis and metabolism consist of many translation and transcription steps, the genes involved in these processes may contribute to the level of estrogen and thereby influence the susceptibility to breast cancer. Among the genes identified, *BRCA1* and *BRCA2* mutations have been reported to be associated with a dominantly inherited increased risk of the disease. However, they only account for about 5% of breast cancer occurrences [2]. This fact leaves the possibility that low-penetrance genetic factors are likely to explain most of disease cases.

Catechol-O-methyltransferase (*COMT*) is an important phase II enzyme involved in the conjugation and inactivation of catechol estrogens [3]. *COMT* is expressed at high levels in a variety of human tissues including liver, kidney, breast, and red blood cells [4]. The *COMT* gene is located on chromosome 22q11 [5]. A G to A transition in the *COMT* gene results in valine to methionine amino acid change in codon 108/158 in the cytosolic/membrane-bound form of the protein. This amino acid change is believed to result in a 3–4-fold decrease in enzymatic activity [6,7]. Since the variant form (Met) has been associated with decreased activity of the *COMT* compared with the wildtype (Val), these two forms are represented as *COMT*-L allele and *COMT*-H allele, respectively. It has been hypothesized that the individuals who inherit the low activity *COMT*-L gene may be at increased risk for breast cancer because of an increased accumulation of the catechol estrogen intermediates [8-11].

The role of COMT Val158Met polymorphism in the development of breast cancer has been investigated in the past decade, with conflicting results. Several studies have previously suggested an association between the COMT Val158Met polymorphism and an increased risk of breast caner [12-14]. However, other studies have failed to confirm such an association [15,16]. Moreover, two meta-analyses investigating the same hypothesis [17,18], quite similar in methods and performed almost at the same time, yielded different conclusions. The exact relationship between genetic polymorphisms of COMT Val158Met and susceptibility to breast cancer has not been entirely established. To clarify the effect of COMT Val158Met on the risk of breast cancer, our study undertakes a meta-analysis of all published case–control observational studies.

## Materials and methods

### Search strategy

Electronic databases PubMed ( http://www.ncbi.nlm.nih.gov/pubmed/), Embase ( http://www.embase.com/) and Cochrane Library ( http://www.thecochranelibrary.com/view/0/index.html) were used to search for all genetic association studies evaluating the *COMT* Val158Met polymorphism and breast cancer risk up to February 2012, the search strategy was based on combinations of “Breast cancer”, “Catechol-O-methyltransferase”, “*COMT*”, “polymorphism”, and “mutation”. No language or country restrictions were applied. All eligible studies were retrieved, and their bibliographies were checked for other relevant publications. Review articles and bibliographies of other relevant studies identified were searched by hand to find additional eligible studies. When multiple publications reported on the same or overlapping data, we chose the most recent or largest population. When a study reported the results on different subpopulations, we treated it as separate studies in the meta-analysis.

### Selection criteria

Studies included in our meta-analysis had to meet the following inclusion criteria: (1) evaluate the association between *COMT* Val108/158Met polymorphism and breast cancer risk; (2) case–control design; (3) sufficient data for estimating an odds ratio (OR) with 95% confidence interval (CI); and (4) studies with full text articles. Studies were excluded if one of the following existed: (1) no control population; and (2) duplicate of previous publication.

### Data extraction

Information was carefully extracted from all eligible publications by two investigators (Xue Qin and Qiliu Peng) independently according to the inclusion criteria listed above. For conflicting evaluation, an agreement was reached following discussion during a consensus meeting with a third reviewer (Aiping Qin). For each study, the following information were collected: First author’s name, year of publication, country, ethnicity of the studied population, total numbers of cases and controls, breast cancer diagnosis criteria, matching criteria, genotyping method, menopausal status, sources of the control population, quality control of genotyping and *P* value for control population in Hardy–Weinberg equilibrium (HWE). We did not define any minimum number of patients to include in our meta-analysis.

### Statistical analysis

Crude odds ratios (ORs) together with their corresponding 95% CIs were used to assess the strength of association between the *COMT* Val158Met polymorphism and breast cancer risk. The pooled ORs were performed for co-dominant model (LL vs. HH, HL vs. HH, and LL vs. HL), dominant model (LL+ HL vs. HH), and recessive model (LL vs. HL+HH), respectively. Departure from the Hardy–Weinberg equilibrium for the control group in each study was assessed using a web-based program ( http://ihg2.helmholtz-muenchen.de/cgibin/hw/hwa1.pl). In subgroup analysis, we evaluated the effect of *COMT* Val108/158Met polymorphism on the susceptibility of *BC* in different population stratified by ethnicity (Caucasian, Asian, and Mixed/other), menopausal status (Pre-, and Post-) and sources of the control population (HB, PB, and FB).

For each genetic comparison, a chi-square-based *Q*-statistic test was used to evaluate the between-study heterogeneity of the studies. If *P* < 0.10, the between-study heterogeneity was considered to be significant, we chose the random-effects model to calculate the OR. Otherwise, when *P* ≥ 0.10, the between study heterogeneity was not significant, then the fixed effects model was used. We also measured the effect of heterogeneity using a quantitative measure, *I*^*2*^ = 100% × (*Q* – *df*)/*Q*[19]. The *I* statistic measures the degree of inconsistency in the studies by calculating what percentage of the total variation across studies is due to heterogeneity rather than by chance [20]. Finally, the overall or pooled estimate of risk (OR) was calculated by a random effects model (DerSimonian–Laird) or a fixed effects model (Mantel–Haenszel) according to the presence (*P* < 0.10 or *I*^*2*^ > 50%) or absence (*P* ≥ 0.10 and *I*^*2*^ ≤ 50%) of heterogeneity, respectively.

Cumulative meta-analysis was conducted to identify the influence of the first published study on the subsequent publications, and the evolution of the combined estimates over time according to the ascending date of publication. To identify potentially influential studies, sensitivity analysis was also performed by excluding the studies without definite diagnostic criteria, the studies without quality control when genotyping and the studies whose genotype frequencies in control populations exhibited significant deviation from the Hardy–Weinberg equilibrium (HWE), given that the deviation may denote bias. The funnel plots and Egger regression asymmetry test were used to assess publication bias. Egger’s test can detect funnel plot asymmetry by determining whether the intercept deviates significantly from zero in a regression of the standardized effect estimates against their precision. A T test was performed to determine the significance of the asymmetry. An asymmetric plot suggested possible publication bias (*P* ≥ 0.05 suggests no bias). All analyses were performed using Stata software, version 10.0 (Stata Corp., College Station, TX, USA).

## Results

### Study characteristics

According to our search criteria, 61 studies relevant to the role of *COMT* Val158Met polymorphism on *BC* risk were identified. Ten of these articles were excluded: one of these articles was a review [21], four were overlapped subjects [22-25], four did not provide allele or genotyping data [26-29], and one was a study concerned with *COMT* 1222 G>A polymorphism [30]. Manual search of references cited in the published studies did not reveal any additional articles. As a result, a total of 51 relevant studies met the inclusion criteria for the meta-analysis [9-16,31-73]. Among them, five of the eligible studies contained data on two different ethnic groups, and we treated them independently [31,51,56,60,69]. Therefore, a total of 56 separate comparisons consisting of 34,358 *BC* patients and 45,429 controls were included in our meta-analysis. The characteristics of the 56 case–control comparisons selected for determining the relationship between *COMT* Val108/158Met polymorphism and risk of *BC* are summarized in Table 1. These 56 comparisons were consisted of 33 Caucasian samples, 18 Asian populations and 5 mixed/other populations. Thirty of the studies were population-based case–control studies and 20 were hospital-based studies, four of these studies [44,54,60,69] presented *COMT* Val158Met polymorphism genotype distributions according to family history (familial-based breast cancer). There were 22 comparisons concerned with *COMT* Val158Met polymorphism and premenopausal BC patients and 27 comparisons concerned with *COMT* Val158Met polymorphism and postmenopausal BC patients (see Table 1). Seventy-one percent (40/56) studies in the present meta-analysis used the golden criteria of “histologically confirmed” or “pathologically conformed” as *BC* diagnosis. Eighty-two (46/56) percent of the control populations matched to *BC* patients with age and 52% (29/56) studies used the classic PCR-RFLP assay to genotype the *COMT* Val158Met polymorphism, about 52% (29/56) of the case–control studies included mentioned the quality control when genotyping. The genotype frequencies of control group in 3 studies were not consistent with HWE [33,41,70]. We could not calculate the *P* value of HWE in two studies [66,73] because they only provided data with dominant model. To remove possible HWE stratification, for each analysis involving any of these 5 studies, sensitivity analysis would be carried out by excluding the studies the genotype frequencies for control group of which deviate from HWE and the studies whose *P* value of HWE in the control group could not be calculated.

**Table 1:** **General characteristics of individual studies in the meta-analysis of *COMT *Val158Met polymorphism and breast cancer**

**Study, year**	**Country**	**Ethnicity**	**No. of cases/controls**	**BC diagnosis**	**Matching criteria**	**Genotyping method**	**Menopausal status**	**Control sources**	**Quality control**	**HWE**^**6**^**(*p *value)**
Lavigne 1997	America	Caucasian	113/114	NR	Age, race	PCR-RFLP	Pre-, Post-	HB	NR	0.862
Thompson 1998	America	Caucasian	281/289	Histologically confirmed	Age, region	PCR-RFLP	Pre-, Post-	PB	NR	0.522
Millikan^a^ 1998	America	Caucasian	389/379	Histologically confirmed	Age, race	PCR-RFLP	Pre-, Post-	PB	Yes	0.916
Millikan^b^ 1998	America	Mixed/other	265/263	Histologically confirmed	Age, race	PCR-RFLP	Pre-, Post-	PB	Yes	0.838
Huang 1999	China	Asian	118/125	Pathologically conformed	NR	PCR-RFLP	Pre-, Post-	HB	NR	0.612
Goodman 2001	America	Caucasian	112/113	Histologically confirmed	Age, race	PCR-RFLP	Mixed	PB	Yes	0.788
Mitrunen 2001	Finland	Caucasian	481/480	Histologically confirmed	NR	PCR-RFLP	Pre-, Post-	PB	NR	0.921
Yim 2001	Korea	Asian	163/163	Histopathologically confirmed	Age	PCR-RFLP	Pre-, Post-	HB	Yes	**0.004**
Jungestrom 2001	Sweden	Caucasian	126/117	NR	Age	PCR-RFLP	Pre-	HB	NR	0.209
Hamajima 2001	Japan	Asian	150/165	Histologically confirmed	NR	PCR-RFLP	Pre-, Post-	HB	NR	0.079
Kocabas 2002	Turkey	Caucasian	84/103	Histologically confirmed	Age	PCR-RFLP	Pre-, Post-	HB	NR	0.227
Comings 2003	America	Caucasian	67/145	NR	Region	PCR-RFLP	Post-	PB	NR	0.335
Wedren 2003	Sweden	Caucasian	1490/1340	NR	Age	DASH	Post-	PB	Yes	0.772
Wu 2003	America	Asian	589/562	NR	Age, race	TaqMan	Mixed	PB	NR	0.646
Tan 2003	China	Asian	250/250	Histopathologically confirmed	Age	PCR-RFLP	Pre-, Post-	HB	NR	0.174
Sazci 2004	Turkey	Caucasian	130/224	Histopathologically confirmed	Age	PCR-RFLP	Pre-	PB	NR	**0.000**
Dunning 2004	UK	Caucasian	2850/1908	NR	Age, region	TaqMan	Post-	PB	Yes	0.232
Hefler 2004	Austria	Caucasian	391/1698	Histologically confirmed	Age, region	Sequencing	Mixed	HB	Yes	0.577
Ahsan 2004	America	Caucasian	313/262	Histopathologically confirmed	Age	LP	Mixed	FB	Yes	0.108
Modugno 2005	America	Caucasian	250/3950	Histopathologically confirmed	NR	TaqMan	Post-	PB	NR	0.391
Lin 2005	China	Asian	99/366	Pathologically conformed	Age, region	PCR-RFLP	Mixed	PB	Yes	0.972
Lin 2005	China	Asian	87/341	Pathologically conformed	Age, region	PCR-RFLP	Mixed	PB	Yes	0.393
Marchand 2005	America	Mixed/other	1339/1370	NR	Age	PCR-RFLP	Post-	PB	NR	0.109
Wen 2005	China	Asian	1120/1191	Pathologically conformed	Age	PCR-RFLP	Pre-, post-	PB	Yes	0.698
Cheng 2005	China	Asian	496/740	Pathologically conformed	Age	NR	Mixed	HB	Yes	0.006
Gaudet^a^ 2006	America	Caucasian	1048/1092	Pathologically conformed	Age	MALDI-TOF	Pre-, post-	PB	Yes	0.853
Gaudet^b^ 2006	Poland	Caucasian	1983/2279	Histopathologically confirmed	Age	TaqMan	Mixed	PB	Yes	0.525
Gallicchio 2006	America	Caucasian	81/1251	Pathologically conformed	NR	TaqMan	Mixed	PB	NR	0.440
Chang 2006	China	Asian	189/321	Histologically confirmed	Age	PCR-RFLP	Mixed	HB	NR	0.068
Onay 2006	Canada	Caucasian	398/372	Pathologically conformed	Age	TaqMan	Pre-	FB	Yes	0.283
Pharoah 2007	UK	Caucasian	2176/2012	NR	NR	TaqMan	Mixed	PB	NR	0.287
Ralph^a^ 2007	America	Caucasian	1626/3286	NR	Age	TaqMan	Pre-, post-	HB	Yes	0.758
Ralph^b^ 2007	America	Caucasian	500/1005	NR	Age	TaqMan	Pre-, post-	HB	Yes	0.549
Akisik 2007	Turkey	Caucasian	114/108	NR	Age	PCR-RFLP	Mixed	NR	NR	0.966
Hu 2007	China	Asian	112/110	Pathologically conformed	Age	Sequencing	Pre-, post-	HB	NR	0.252
Takata 2007	America	Mixed/other	325/250	Mammographically examed	Age	PCR-RFLP	Pre-, post-	PB	NR	0.104
Onay^a^ 2008	Canada	Caucasian	1217/714	Pathologically conformed	Age	TaqMan	Mixed	FB	Yes	0.832
Onay^b^ 2008	Finland	Caucasian	708/549	Pathologically conformed	Age	TaqMan	Mixed	FB	Yes	0.676
Justenhoven 2008	Germany	Caucasian	606/622	NR	Age	MALDI-TOF MS	Mixed	PB	Yes	0.654
He 2009	America	Caucasian	1212/1683	Pathologically conformed	Age	TaqMan	Mixed	HB	Yes	0.850
Reding 2009	America	Caucasian	891/878	NR	Age	TaqMan	post-	PB	Yes	0.606
GENICA 2009	Germany	Caucasian	3144/5481	Histologically conformed	Age, region	MALDI-TOF MS	post-	PB	Yes	0.094
Yadav 2009	India	Asian	154/166	NR	Region	PCR-RFLP	Pre-, post-	HB	NR	0.570
Shrubsole 2009	China	Asian	1093/1169	Pathologically conformed	Age	PCR-RFLP	Pre-, post-	PB	Yes	—
Sangrajrang 2009	Thailand	Asian	565/486	Histologically conformed	NR	TaqMan	Mixed	HB	Yes	0.610
Mónica 2010	Mexico	Caucasian	91/94	Pathologically conformed	Age, education	PCR-RFLP	Pre-, post-	HB	NR	0.669
Syamala^a^ 2010	India	Asian	219/367	Histologically conformed	Age	PCR-RFLP	Mixed	PB	NR	0.183
Syamala^b^ 2010	India	Asian	140/367	Histologically conformed	Age	PCR-RFLP	Mixed	FB	NR	0.183
Peterson 2010	America	Caucasian	1584/1416	Pathologically conformed	Age	TaqMan	Mixed	PB	Yes	**0.026**
Delort 2010	France	Caucasian	910/1000	Pathologically conformed	Age	TaqMan	Mixed	PB	Yes	0.230
Wang 2011	China	Asian	400/400	Histopathologically conformed	Age	Sequencing	Pre-, post-	PB	Yes	0.389
Naushad 2011	India	Asian	212/233	Histopathologically conformed	NR	PCR-RFLP	Mixed	HB	NR	0.201
Cribb 2011	Canada	Caucasian	207/621	Histopathologically conformed	Age	PCR-RFLP	Mixed	HB	NR	0.208
Cerne 2011	Slovenia	Caucasian	530/270	NR	Age	TaqMan	post-	HB	Yes	0.903
Lajin 2011	Syria	Mixed/other	135/107	Pathologically conformed	Age	PCR-RFLP	Pre-, post-	PB	NR	0.887
Santos 2011	Brazil	Mixed/other	62/62	Pathologically conformed	Age	PCR-RFLP	Pre-, post-	PB	NR	—

*PB* Population-based *FB* family-based, *HB* hospital-based, *HWE* Hardy–Weinberg equilibrium, *NR* not reported, *Pre-* premenopausal, *Post-* postmenopausal, *PCR-RFLP* PCR-based restriction fragment length polymorphism, *MALDI-TOF MS* matrix assisted laser desorption/ionization time-of-flight mass spectrometry, *LP* Luorescence polarization.

^a, b^ They were two different case–control studies in one publication.

### Quantitative synthesis of data

**Figure 1:**
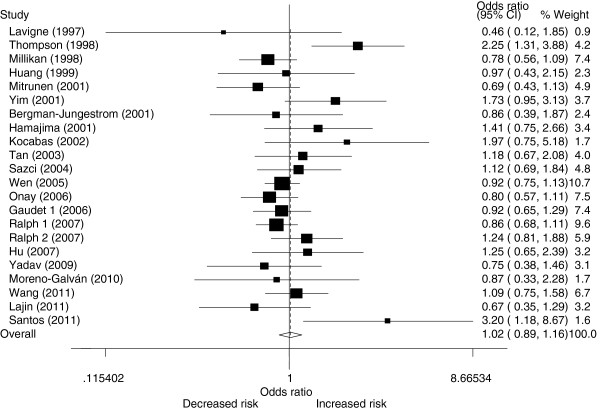
**OR and 95% CI of individual studies and pooled data for the association between the *COMT *Val158Met polymorphism and *BC *in premenopausal populations using a random-effect model (dominant model LL+HL vs. HH).**

**Figure 2:**
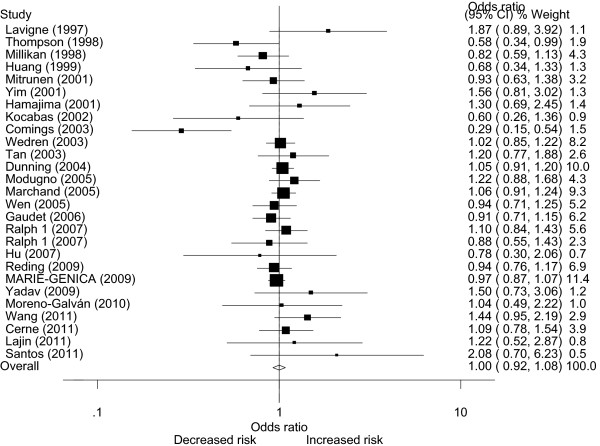
**OR and 95% CI of individual studies and pooled data for the association between the *COMT *Val158Met polymorphism and *BC *in postmenopausal populations using a random-effect model (dominant model LL+HL vs. HH).**

**Figure 3:**
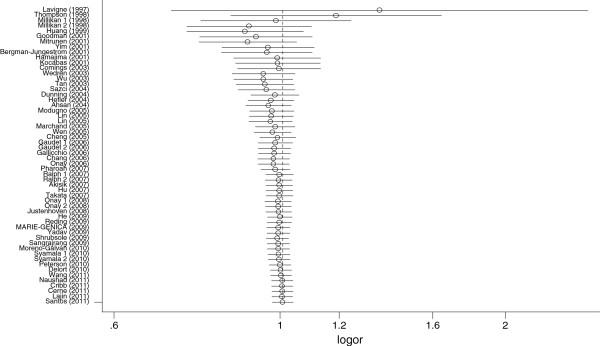
**Cumulative meta-analysis of the association between *COMT *Val158Met polymorphism and breast cancer susceptibility risk of the overall populations using a random effects model (dominant model LL+HL versus HH).** Each study was used as an information step. The vertical dotted line is the summary odds ratio. Bars, 95% confidence interval (CI).

The pooled ORs along with their 95% CIs and the results of the heterogeneity test are presented in detail in Table 2. Overall, no significant associations between *COMT* Val158Met polymorphism and breast cancer susceptibility were observed in all genetic models when all the eligible studies were pooled into the meta-analysis. No significant associations were found for LL versus HH (OR = 0.999, 95% CI 0.0.925–1.078; *I*^*2*^*=*55.0 and *P* = 0.000 for heterogeneity), HL versus HH (OR = 1.005, 95% CI 0.959–1.052; *I*^*2*^*=*27.1 and *P* = 0.038 for heterogeneity), LL versus HL (OR = 0.983, 95% CI 0.926–1.045; *I*^*2*^*=*44.4 and *P* = 0.000 for heterogeneity), recessive model LL versus HL+HH (OR = 0.988, 95% CI 0.929–1.050; *I*^*2*^*=*51.3 and *P* = 0.000 for heterogeneity) and dominant model LL+HL versus HH (OR = 1.001, 95% CI 0.954–1.051; *I*^*2*^*=*41.0 and *P* = 0.001 for heterogeneity). Next, the effect of *COMT* Val158Met polymorphism on breast cancer risk was evaluated according to ethnicity, menopausal status (Figure 1; Figure 2) and sources of controls. Similarly, no significant association was found in any of the genetic models. We further conducted a meta-analysis after the five studies [33,41,66,70,73] whose genotype frequencies significantly deviated from HWE or whose *P* values of HWE in the control population unable to be calculated were excluded. The results were not materially changed in any genetic models. Sensitivity analysis by excluding the studies without definite diagnostic criteria and the studies without quality control when genotyping did not alter the pattern of the results. Cumulative meta-analysis was performed for dominant model LL +LH versus HH in the overall populations. In the overall populations, the random effects odds ratio was always insignificantly larger or smaller than 1. It changed little from around 0.998 after the year 2007 (Figure 3), indicating the stability of the association.

**Table 2:** **Meta-analysis of the *COMT *Val158Met polymorphism on *BC *susceptibility**

**Comparison**	**Population**	**Sample size**		**No. of studies**	**Test of association**			**Mode**	**Test of heterogeneity**		
		**Case**	**Control**		**OR**	**95% CI**	***P *value**		***χ***^***2***^	***P *value**	***I***^***2***^
LL vs. HH	Overall	17,223	23,069	54	0.999	0.925-1.078	0.976	R	117.76	0.000	55.0
	Caucasian	12,942	18,066	32	0.960	0.897-1.028	0.240	R	49.28	0.020	37.1
	Asian	3,009	3,790	17	1.243	0.942-1.641	0.125	R	54.34	0.000	70.6
	Pre-	2,095	2,523	21	1.049	0.825-1.334	0.697	R	48.22	0.000	58.5
	Post-	7,215	10,138	26	0.982	0.875-1.102	0.757	R	45.40	0.008	44.9
	PB	17,223	23,069	28	0.999	0.925-1.078	0.381	R	48.00	0.008	43.7
	HB	3,800	6,169	20	1.151	0.946-1.402	0.160	R	58.86	0.000	67.7
	FB	1,351	1,140	5	0.848	0.712-1.010	0.064	F	4.43	0.351	9.7
HL vs. HH	Overall	22,589	33,568	54	1.005	0.959-1.052	0.845	R	72.70	0.038	27.1
	Caucasian	19,059	25,912	32	0.999	0.958-1.042	0.968	F	30.14	0.510	0.0
	Asian	4,525	5,781	17	1.052	0.923-1.200	0.448	R	36.85	0.002	56.6
	Pre-	3,204	3,877	21	0.962	0.871-1.062	0.440	F	27.59	0.119	27.5
	Post-	10,480	14,476	26	1.009	0.954-1.067	0.762	F	33.83	0.112	26.1
	PB	17,648	22,679	28	0.987	0.945-1.030	0.547	F	3.60	0.463	0.0
	HB	5,751	9,128	20	1.075	0.966-1.195	0.187	R	33.89	0.019	43.9
	FB	2,102	1,674	5	0.950	0.824-1.094	0.476	F	30.98	0.272	12.9
LL vs. HL	Overall	23,594	31,759	54	0.983	0.926-1.045	0.586	R	95.26	0.000	44.4
	Caucasian	19,579	27,208	32	0.954	0.911-1.001	0.055	F	36.02	0.245	13.9
	Asian	2,538	3,135	17	1.170	0.895-1.528	0.251	R	49.83	0.000	67.9
	Pre-	2,507	3,200	21	1.060	0.851-1.320	0.606	R	49.32	0.000	59.4
	Post-	10,243	14,548	26	0.969	0.915-1.025	0.271	F	32.47	0.271	23.0
	PB	16,437	21,768	28	0.969	0.909-1.032	0.324	R	39.76	0.054	32.1
	HB	4,973	8,203	20	1.060	0.902-1.245	0.478	R	48.71	0.000	61.0
	FB	2,099	1,714	5	0.882	0.769-1.012	0.073	F	4.37	0.358	8.6
LL vs. HL+HH	Overall	34,358	45,429	56	0.988	0.929-1.050	0.702	R	108.88	0.000	51.3
	Caucasian	25,790	35,593	32	0.956	0.909-1.006	0.081	R	43.54	0.067	28.8
	Asian	5,770	7,552	17	1.204	0.927-1.564	0.164	R	52.91	0.000	69.8
	Pre-	3,903	4.800	21	1.053	0.855-1.297	0.627	R	49.44	0.000	59.5
	Post-	13,969	19,581	26	0.980	0.901-1.065	0.629	R	37.85	0.048	33.9
	PB	24,205	31,307	30	0.966	0.906-1.030	0.290	R	45.36	0.015	40.5
	HB	7,262	11,750	20	1.098	0.934-1.289	0.257	R	54.38	0.000	65.1
	FB	2,776	2,264	5	0.877	0.760-1.013	0.074	F	4.62	0.328	13.5
LL+HL vs. HH	Overall	34,358	45,429	56	1.001	0.954-1.051	0.953	R	93.20	0.001	41.0
	Caucasian	25,790	35,593	32	0.982	0.944-1.022	0.369	F	37.71	0.189	17.8
	Asian	5,770	7,552	17	1.072	0.952-1.208	0.253	R	42.65	0.001	60.1
	Pre-	3,933	4.839	22	1.016	0.890-1.160	0.815	R	33.81	0.038	37.9
	Post-	14,001	19,604	27	1.001	0.924-1.084	0.987	R	40.17	0.038	35.3
	PB	24,205	31,307	30	0.975	0.924-1.028	0.343	R	42.89	0.047	32.4
	HB	7,262	11,750	20	1.091	0.978-1.216	0.118	R	39.26	0.004	51.6
	FB	2,776	2,264	5	0.914	0.799-1.044	0.186	F	3.81	0.432	0.0

*OR* odds ratio, *CI* confidence intervals, *R* random effects model, *F* fixed effects model, *PB* Population-based study, *HB* Hospital-based study, *FB* Familial-based study, *Pre-* Premenopausal, *Post-* Postmenopausal.

### Publication bias

**Figure 4:**
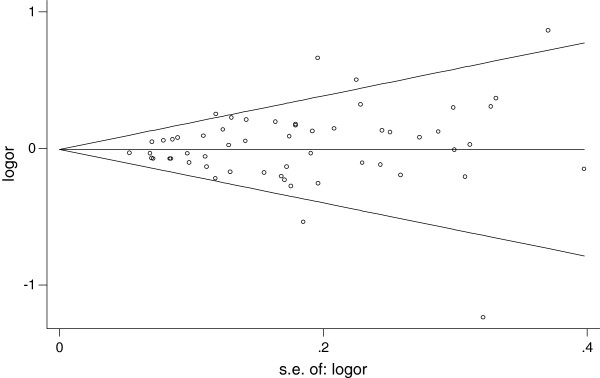
**Funnel plots for publication bias in the studies of the meta-analysis on the association between *COMT *Val158Met polymorphism and breast cancer risk of the overall populations (dominant LL+HL versus HH).**

Begg’s funnel plots and Egger’s tests were performed to assess publication bias. The shapes of the funnel plots revealed no obvious asymmetry (Figure 4). The Egger’s test was then used to statistically assess funnel plot symmetry. The results suggested no evidence of publication bias (*t* = 0.94 and *P* = 0.352 for dominant model). The results indicated that the results of these meta-analyses are relatively stable and that publication bias is unlikely to affect the results of the meta-analyses.

## Discussion

Estrogens, estrone, and estradiol are catabolized to catechol estrogens. Estrogen metabolites, such as 4-hydroxyestrone and 4-hydroxyestrone, shown to be involved in breast carcinogenesis [74]. Catechol-O-methyltransferase (*COMT*) catalyzes the O-methylation of these carcinogenic estrogens to methoxyes tradiols and methoxyestrones. In the *COMT* gene, a G to A transition results in an amino acid change (Val/Met) at codon 108 of soluble *COMT* and codon 158 of membrane-bound *COMT*. This amino acid change is believed to result in a 3–4-fold decrease in enzymatic activity [6,7]. It has been hypothesized that individuals who inherit the low activity COMT gene may be at increased risk for breast cancer because of an increased accumulation of the catechol estrogen intermediates. The potential association between the *COMT* Val108/158Met polymorphism and the risk of subsequent *BC* has evoked a huge interest from clinicians, scientists, and the public. During the past few years a large number of studies with case–control design have been carried out to investigate this topic but consistent results have not been reported. We therefore conducted a meta-analysis of the evidence obtained from all published studies in order to elucidate and provide a quantitative reassessment of the association. To our knowledge, this is the most comprehensive meta-analysis to date to evaluate the association between *COMT* Val108/158Met polymorphism and breast cancer risk.

We did not observe a positive relationship between *COMT* Val108/158Met polymorphism and breast cancer risk either overall or among subgroups of women defined by ethnicity, menopausal status or sources of the control population. In previous studies, overall the findings were inconsistent. Lavigne et al. observed a large increase in the risk of breast cancer among postmenopausal obese women carrying the *COMT*-LL genotype, and an inverse association among premenopausal women with the relative risk (RR) for *COMT*-LL stronger among postmenopausal women with high BMI [9]. Thompson et al. reported positive associations for the *COMT*-HL and *COMT*-LL genotypes among premenopausal women and found that modification of RRs by BMI was highest among premenopausal women with a high BMI [10]. A comprehensive study of the entire estrogen-metabolizing pathway (*CYP17, CYP1A1, COMT*) also reported that breast cancer is only associated with the low activity COMT genotype in women with a high BMI and that the *COMT*-LL genotype was strongly associated with breast cancer risk, with an adjusted OR of as high as 4.02 [12]. In contrast to the other studies but in line with the findings of the current study, Lajin et al. did not observe any association between one or two copies of the *COMT*-L allele and breast cancer risk, and did not find strong modification of RR estimates by menopausal status [72]. In an effort to shed some light on the impact of *COMT* Val108/158Met polymorphism on breast cancer risk, two previous meta-analyses [17,18] were conducted almost at the same time to explore the relationship between *COMT* Val108/158Met polymorphism and breast cancer. Ding et al. [18] examined the effect of *COMT* Val158Met polymorphism on breast cancer risk by combining results in meta-analysis. They concluded that *COMT* Val158Met polymorphism was significantly associated with increased breast cancer risk in European population. However, Mao et al. [17] did not find any relationship between *COMT* Val158Met polymorphism and breast cancer risk in any genetic models including among Caucasian, Asian, premenopausal, and postmenopausal women in their meta-analysis, which was consistent with the findings of our study. The discrepancy in previously reported findings was most probably because that the previous studies with relatively small sample size may have insufficient statistical power to detect the exact effect or may have generated a fluctuated risk estimate. However, in our study, large number of cases and controls were pooled from all published studies, which greatly increased statistical power of the analysis and provided enough evidence for us to draw a safe and reliable conclusion.

Heterogeneity is a potential problem that may affect the interpretation of the results. The present meta-analysis showed that there was large heterogeneity between studies (table 2). Common reasons for heterogeneity may include differences in the studied populations (e.g., ethnicity, menopausal status), or in methods (e.g., genotyping), or in sample selection (e.g., source of control populations), or it may be due to interaction with other risk factors (e.g., *BRCA* variants). Finding of the source of heterogeneity is one of the most important goals of a meta-analysis. Therefore, we stratified the studies according to ethnicity, source of control subjects of the studies, and menopausal status. Subsequent subgroup analysis stratified by ethnicity, source of control subjects, and menopausal status identified large heterogeneity as well, indicating that menopausal status, ethnicity or source of control subjects contributed little to the existence of overall heterogeneity. Unfortunately, our study had insufficient information for subgroup analysis to detect whether the variants in *BRCA* gene might be great sources of heterogeneity. We found that in three studies [33,41,70] the genotypic frequencies showed significant deviation from the expected frequencies based on Hardy–Weinberg equilibrium and two studies [66,73] provide insufficient data for calculating P value of HWE in the control populations. Excluding these five studies did not alter the heterogeneity between studies. However, when heterogeneity between the studies exists, the results could be interpreted in the context of cumulative meta-analysis, which provides a measure of how much the genetic effect changes as more data accumulate over time [75]. In our study, the results of cumulative meta-analysis for dominant model LL+HL versus HH showed stability in pooled odds ratio after the year 2007 in the overall populations, which provide evidence for drawing safe conclusion about the insignificant association between *COMT* Val158Met polymorphism and breast cancer risk.

Some limitations of this meta-analysis should be acknowledged. First, some studies found significant associations between *COMT* Val108/158Met polymorphism and breast cancer risk in several subgroups of populations, such as associations among postmenopausal women with a low body mass index (BMI) [10,11], a high BMI [9] or women at young ages [11]. It is difficult for a meta-anlysis to derive such specific associations because the results from previous studies were not presented in a uniform standard. Second, our results were based on unadjusted estimates and a more precise analysis should be carried out if individual data were available, this would allow for adjustment by other covariates including age, BMI, ethnicity, lifestyle, and environmental factors. Third, all of the studies were performed in Asian and Caucasian populations. Further studies are needed in other ethnic populations because of possible ethnic differences of the *COMT* polymorphisms. In spite of these, our present meta-analysis also had some advantages. First, substantial number of cases and controls were pooled from all publications concerned with *COMT* Val158Met polymorphism and BC risk, which greatly increased statistical power of the analysis and provided enough evidence for us to draw a safe conclusion. Second, the quality of case–control studies included in this meta-analysis was satisfactory according to our selection criteria. Third, no publication bias was detected in this meta-analysis, which indicated that the pooled results of our study should be reliable.

In conclusion, this meta-analysis suggests that the *COMT* Val158Met polymorphism may not be associated with breast cancer risk. However, it is necessary to conduct large sample studies using standardized unbiased genotyping methods, homogeneous breast cancer patients, and well-matched controls. Moreover, gene-gene and gene-environment interactions should also be considered in the analysis. Such studies taking these factors into account may eventually lead to a better, more comprehensive understanding of the association between *COMT* Val158Met polymorphism and BC risk.

## Abbreviations

BC: Breast cancer; HWE: Hardy–Weinberg equilibrium; OR: Odds ratio; CI: Confidence interval; COMT: Catechol-O-methyltransferase; BMI: Body mass index; PB: Population-based; FB: Family-based; HB: Hospital-based; Pre: Premenopausal; Post: Postmenopausal; PCR-RFLP PCR: based restriction fragment length polymorphism; MALDI-TOF MS: matrix assisted laser desorption/ionization time-of-flight mass spectrometry; LP: Luorescence polarization.

## Competing interest

The authors declared that they have no conflict of interest in relation to this study.

## Authors’ contributions

All authors have read and approved the final files for this manuscript.
